# RESEARCHERS BEWARE: CAUTIONARY TALES OF FRAUDULENT RESEARCH PARTICIPANTS AND WHAT TO DO ABOUT THEM

**DOI:** 10.1093/geroni/igad104.1174

**Published:** 2023-12-21

**Authors:** Justine Sefcik, Harleah Buck

## Abstract

This symposium presents a series of cases where gerontological researchers identified fraudulent participants and bots engaging in their studies. These presentations describe the complex nature of participants misrepresenting themselves and being creative to enroll in studies for financial incentives. Dr. Sefcik shares how a snowball sample led to participants enrolling in a study and misrepresenting themselves during virtual qualitative interviews. Dr. Boon illuminates how Facebook recruitment led to bot responses and steps taken to identify if participants were real. Dr. Frechman reveals how recruitment on social media platforms and email distribution lists led to bot attacks of the study survey. Dr. Carpenter explains a study involving a multi-methods approach in which a bot completed several surveys and an interviewee gave nonsensical responses. Dr. Wallace explains two types of fraudulent activity occurring, the first with bots completing an online survey and the second with deception during interviews. All presenters discuss their experiences of suspecting fraudulent research participation, approaches on how they verified participants, action steps to address misrepresentation, processes put in place to uphold the integrity of their studies, and tips to mitigate future fraud. This is a Nursing Care of Older Adults Interest Group Sponsored Symposium.

